# Walking for Health

**Published:** 1892-11

**Authors:** 


### WALKING FOR HEALTH.

Few things, if any, are so effectual in building up and sustaining the physical organization as walking, if resolutely and judiciously followed. It is a perfect exercise. It taxes the entire system. When you walk properly, every member and muscle, every nerve and fiber has something to do. The arms swing backward and forward, keeping step, as it were, with the legs; the chest expands and contracts as the lungs fill and discharge ; the drummer-boy pulse beats a tune for the march ; the legs curve and straighten ; the feet rise and fall, while the head rides over all—but not as a deadhead. Every sense it has is employed, every faculty alert. The nostrils expand to quaff the breeze ; the ears turn to every sound ; the eyes roll in their sockets, sweeping from left to right, from earth to sky ; the brain is at work through all its parts. Progress under such conditions is the very eloquence of physical motion. What is the effect ? The flesh is solidified ; the lungs grow strong and sound ; the chest enlarges ; the limbs are rounded out ; the tendons swell and toughen ; the figure rises in height and dignity, and is clothed with grace and suppleness. Hunters, who walk much, are tall and straight, while sailors, who walk scarcely at all, are low and squat. The whole man is developed, not the body merely. The mind is broadened by the contemplation of creation's work, the soul is enlarged, the imagination brightened, the spirits cheered, the temper sweetened. The moral forces are strengthened equally with the physical. A loftier, reverential feeling is awakened, if not a profound religious sentiment. No one who rightly walks the fields and groves or climbs the heights beneath the heavenly dome, with its blazing sun by day and its moon and countless stars by night, but is irresistibly drawn toward the infinite as "he looks through nature up to nature's God."

